# Neurological and Epigenetic Implications of Nutritional Deficiencies on Psychopathology: Conceptualization and Review of Evidence

**DOI:** 10.3390/ijms160818129

**Published:** 2015-08-05

**Authors:** Jianghong Liu, Sophie R. Zhao, Teresa Reyes

**Affiliations:** School of Nursing, University of Pennsylvania, 418 Curie Blvd., Philadelphia, PA 19104, USA; E-Mails: sophie.r.zhao@vanderbilt.edu (S.R.Z.); reyestm@mail.med.upenn.edu (T.R.)

**Keywords:** molecular epigenetics, nutrients, brain dysfunction, gene–environment interactions, behavior problems, psychopathology, neurotoxicity

## Abstract

In recent years, a role for epigenetic modifications in the pathophysiology of disease has received significant attention. Many studies are now beginning to explore the gene–environment interactions, which may mediate early-life exposure to risk factors, such as nutritional deficiencies and later development of behavioral problems in children and adults. In this paper, we review the current literature on the role of epigenetics in the development of psychopathology, with a specific focus on the potential for epigenetic modifications to link nutrition and brain development. We propose a conceptual framework whereby epigenetic modifications (e.g., DNA methylation) mediate the link between micro- and macro-nutrient deficiency early in life and brain dysfunction (e.g., structural aberration, neurotransmitter perturbation), which has been linked to development of behavior problems later on in life.

## 1. Introduction

Increasing evidence has shown that interactions between genetics and environmental factors can modify the physiological response to nutrition [1]. Genetic effects could account for much of the heterogeneity among the population in terms of nutrient intake and personal food preferences. For example in a population-based, twin design study, it was recently confirmed that genetic influences and non-shared environment account for a significant portion of the total energy and macronutrient intake—almost half of the variance in total energy, macronutrients and minerals [2]. The implications of interaction between genetics and environmental factors on nutrition can be further expanded to a variety of physical and mental health outcomes. One particularly interesting area of study is the neurological and epigenetic consequences of nutritional factors on psychopathologies.

For decades, efforts from both the research and clinical communities have been largely unsuccessful in reducing the incidence of psychopathologies, for example childhood antisocial externalizing behavior, adolescent delinquency, as well as adult violent act. One possible explanation is that these efforts predominantly take into account psychosocial factors [3,4,5], while overlooking the role of biological factors, such as nutrition deficiency, in the development of childhood externalizing behaviors and adult antisocial, violent, and criminal behavior [6,7]. In the past decade, studies have begun to recognize the role that nutrition plays in the development of these types of behavior [8,9,10].

Altered brain development has been identified as a potential mechanism [11] through which early nutritional deficits can lead to externalizing behaviors in children, adolescents, and adults. In addition to the environmental causes of nutrition deficiency (e.g., decreased availability, lack of parental knowledge about nutrition planning, *etc.*), genetic variation represents another important facet in diet-psychopathology frameworks. A growing number of studies are beginning to explore the joint effects of genetics and diet on various health outcomes, in which nutritional factors can lead to behavioral outcomes through perturbation of biological pathways, such as growth factors like Brain-derived neurotrophic factor (BDNF) linked to early brain development as well as the synthetic pathways for neurotransmitters [12]*.* More recently, Naninck *et al.* [13] have found that maternal care, stress, perinatal nutrition can alter stress hormones and specific key nutrients during critical brain development periods and act synergistically to program brain structure and function. While there has been increasing evidence that nutrition plays a vital role in linking environmental and genetic factors in health outcomes such as cancer, the role of nutrition in the gene regulation (such as epigenetic modifications) of the development of psychopathological outcomes has received less attention.

The purpose of this paper is to propose a conceptual framework in which the relationship between nutrition deficits and psychopathology is mediated through the interrelated mechanisms of epigenetic modifications and changes in brain development. An overview of the empirical research on nutrition deficiency as a risk factor for psychopathological behavior will be presented briefly, then the focus of the manuscript will be given to the presentation of epigenetic factors and changes in brain structure and function as mechanisms which link nutrition deficiency to psychopathology.

## 2. Overview of the Framework

**Figure 1:**
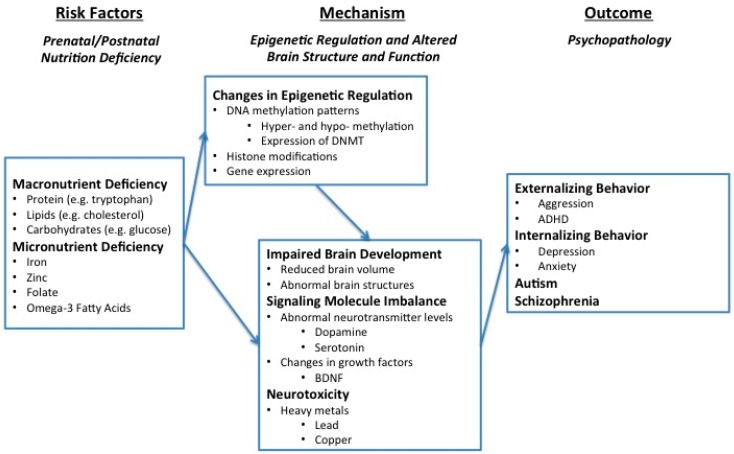
Conceptual Framework of Epigenetic and Altered Brain Structure as Mechanisms of Psychopathology.

The conceptual framework for the nutrition–psychopathology link is depicted in Figure 1. Briefly, the first component, nutrition deficiency, can be attributed to either environmental or genetic risk factors during the prenatal and postnatal periods and is considered, in this framework, to be a risk factor for psychopathological outcomes later in life. In the second component, both macro- and micro-nutrition deficiencies predispose individuals to psychopathology through two interrelated mechanisms: epigenetic changes and altered brain structure and function. More specifically, nutrition deficiency has been linked to important epigenetic changes in the brain such as altering DNA methylation patterns via DNA methyltransferases (DNMTs), histone modifications, and gene expression. Furthermore, epigenetic changes can lead to psychopathology through modifying brain structure and function by at least three routes: (1) changing brain growth and development; (2) disturbing the biochemical processes of signaling molecules; and (3) increasing the toxic effects of neurotoxicants [14,15,16]. Following is a detailed discussion of each component.

## 3. Macro- and Micro-Nutrient Deficiency Are Risk Factors for Psychopathology

Nutrients are normally divided into two categories: macronutrients and micronutrients [17]. Macronutrients often refer to proteins, carbohydrates, fats, macro minerals, and water. Micronutrients, on the other hand, refer to vitamins and trace minerals the body needs daily in amounts on the scale of micrograms to milligrams. Nutrition deficiency can occur at both the macro- and the micro-level, though it is more common for several nutrition deficiencies to exist simultaneously. Studies have indicated that both types of nutrition deficiency are associated with increased behavior problems [6,8,18]. Given the same dietary intake, the effect of nutrient deficiencies varies among individuals based on their body’s ability to utilize specific nutrients. This phenomenon is referred to as bioavailability, which is defined as the proportion of an ingested nutrient or drug that is actually absorbed into the bloodstream [19]. The bioavailability of nutrients is therefore greatly influenced by both genetic and environmental factors. For example, low (or high) absorption from the gastrointestinal tract can be due to genetic program or the effects of exogenous food inhibitor/enhancers [20]. Further, any genetic difference, such as hormonal differences, that affects macro- or micro-nutrient metabolism can also contribute to these observed individual difference [21].

### 3.1. Macro-Nutrition Deficiency and Psychopathology

Protein, fat, and glucose deficits have all been linked to behavioral problems. One main type of macro-nutrition deficiency is protein-energy nutrition deficiency (PEM), or protein-calorie nutrition deficiency. As early as the 1970s, the link between protein deficiency and aggressive behavior has been observed in rats [22]. Recent basic science research revealed that rats with prenatal protein nutrition deficiency exhibited an abnormal locomotor activity rhythm [23] and that rats fed a low-protein diet during early postnatal periods show increased aggressive behavior, as well as impaired learning, retention, and increased impulsiveness [24,25]. Positive associations have also been found between low serum cholesterol and a number of behavioral problems in humans, including antisocial personality disorder and violent and suicidal behavior [26]. Similarly, low non-oxidative glucose metabolism has been found to be a predictor of recurrent violent behavior in humans [27].

Amino acids such as tryptophan have also been strongly implicated in the development of aggressive and violent behavior. Both monkeys and rats fed diets depleted of tryptophan become more aggressive than controls, while diets high in tryptophan reduced aggressive behavior [28]. Furthermore, since tryptophan is the biochemical building block of the neurotransmitter serotonin, diets low in tryptophan could contribute to low level of serotonin found in impulsive and violent offenders [12,29,30,31].

### 3.2. Micro-Nutrition Deficiency and Psychopathology

A number of studies have shown an influence of micronutrients on the development of aggressive, violent, antisocial, and criminal behavior in humans [9,10,32]. Both iron and zinc are important trace metals that are essential for good nutrition and for maintaining brain homeostasis. Many groups have reported the effects of dietary iron and zinc on both brain and behavioral functioning [33,34,35]. Observational studies have found that iron deficiencies are found in aggressive and conduct disordered children [30,36,37].

In a longitudinal cohort study researchers found a dose-response relationship between micronutrient nutrition deficiency (specifically deficiencies in zinc, iron, and vitamin B) at 3 years of age, and externalizing behavior problems across childhood and into adolescence [8]. Zinc deficiency has also been correlated with hyperactivity and Attention Deficient Hyperactivity Disorder (ADHD), in that plasma zinc levels may affect information processing in ADHD children [38]. Arnold and DiSilvestro [39] also reported lower zinc tissue levels in blood serum, red blood cells, hair, urine, and nails in children with ADHD.

Folate deficiency during gestation is also linked to neurobehavioral outcomes of children. Children of mothers with prenatal folate deficiency were at higher risk for emotional problems, especially compared to mothers who started folate supplements periconceptually [40]. A population-based study from in Norway also found that prenatal folic acids supplements could lower the risk for autism disorder [41]. Furthermore, there are also indications of abnormal folate status in patients diagnosed with schizophrenia and neural tube defects from birth cohorts exposed to famine during gestation [42].

Omega-3 fatty acids deficiency has also been hypothesized as an agent in depression, memory problems, mood swings, and many other neurological conditions. Children lacking sufficient amounts of omega-3 fatty acids have been found to present with hyperactivity, learning disorders, and behavioral problems [43,44]. Furthermore, a more recent interventional study showed that omega-3 fatty acid supplementation produced a sustained reduction in behavioral problems of children, including externalizing behavior [45].

Studies have also suggested that nutritional deficits alone may not be entirely responsible for behavior problems. Rather, it may be the interactions between nutrient levels and environmental toxicants, such as heavy metals, which predisposes individuals to antisocial behavior [46,47]. Specifically, low mineral levels may exacerbate the effects of environmental toxicity. For example, adding calcium to the diet can in fact decrease the toxic effects produced by lead exposure [48,49]. Similarly, Masters *et al.* [50] reported that animals fed a diet high in manganese (classified as a toxic heavy metal in high doses) do not exhibit high levels of blood manganese when the diet also contains adequate amounts of calcium. Lead exposure has also been linked to externalizing behavior problems in children. Needleman *et al.* [51] found that increased bone lead concentrations in juveniles was associated with delinquency. We have also found that increased blood lead concentrations in school children are correlated with aggression and attention problems [52].

## 4. Mechanisms Mediating Nutrition Deficiency and Psychopathology: Epigenetics and Altered Brain Structure and Function

As we describe above, the strong connection between nutrition deficiency and psychopathology has been supported by numerous studies. However, the mechanisms by which nutrition deficiency might cause psychopathology are not well understood. We hypothesize two interrelated processes as potential mechanisms: epigenetic modifications and brain dysfunction.

### 4.1. Nutrient Deficiency Alters Epigenetic Processes, Particularly in Early Development

The field of epigenetics involves studying modifications to the genome that can drive changes in gene expression; changes that occur without mutations to the underlying DNA sequences [53]. Some of these modifications may be heritable to subsequent generations, though the extent to which that occurs in humans is questioned, particularly given the extensive epigenetic reprogramming that occurs in early embryogenesis [54,55,56]. Different cell types display distinct gene expression patterns that are influenced by epigenetic modifications highly responsive to environmental and developmental signals. Epigenetic modifications include DNA methylation, chromatin alterations, and a number of recently discovered RNA factors. The epigenetic programming of gene expression is particularly sensitive to nutrition deficiency in the prenatal period and early childhood. A review by McGowan *et al.* [57] describes how diet, along with other environmental influences that occur during pregnancy, can affect epigenetic changes that alter how the nervous system develops. Recently, it was reported that maternal underweight (which is likely driven by nutrient deficiency) was associated with methylation differences in neonatal blood samples [58]. The use of animal models allows for the analysis of brain-specific epigenetic changes, and Pogribny *et al.* [59] found that neurons in adult rat brain underwent widespread epigenetic modifications in response to a folate-deficient diet fed from weaning into adulthood. While early development has been the focus of most studies examining nutrient deficiency and epigenetic modifications, there is some evidence that changes in epigenetic marks can happen during adulthood, at least in the periphery. For example, an increase in muscle PPARGC1α methylation was observed in response to a 36 h fast [60]. Lastly, there is evidence suggesting a level of heritability of epigenetic modifications between generations. In response to undernourishment, both a metabolic phenotype as well as altered gene expression were paternally transmitted to the F2 generation [56], even though alterations in DNA methylation which were seen in the F1 generation did not persist. Together, these studies suggest that the environmentally sensitive epigenetic mechanisms that alter neurological functions can be both determinant (set during development and/or inherited) and possibly dynamic (responsive to acute nutrient changes in adulthood). Because nutrition deficiency can precipitate changes like these in the brain, epigenetic modifications within the brain need to be considered as a mediator between nutrition deficiency and the development of brain dysfunction and psychopathology phenotypes.

#### 4.1.1. Influence of Nutrition on DNA Methylation, Histone Modification, and Gene Expression

While there are numerous epigenetic marks that could potentially be affected by nutrient deficiency, DNA methylation has received the most attention. DNA methylation at critical sites (e.g., regulatory regions such as the promoter) is typically thought to silence gene expression by inhibiting transcription (e.g., inhibiting binding of activational transcription factors), however the relationship between DNA methylation and RNA transcription is not always straightforward [61]. This DNA modification is catalyzed by DNA methyltrasnferases that transfer methyl groups from *S*-adenosylmethionine (SAM) to the 5′ position on cytosine bases [62].

The influence of diet on DNA methylation may be direct in that the SAM feedstock for methylation is derived, in part, from dietary methyl intake. The amino acid methionine is a major source of dietary methyl groups, in addition to other dietary sources including choline (an important precursor to the neurotransmitter acetylcholine), folic acid, and vitamin B12 [63]. In a study involving rural Gambian women, who experience season nutrition changes, there were significant methylation changes on epialleles of offspring depending on the time of conception, based on differences in methyl-donor nutrient intake [64]. Additionally, a second potential mechanism whereby diet could alter DNA methylation involves direct effects on the expression of the DNA methylation machinery, namely the DNA methyltransferase system, as early life protein restriction [65] as well as α-linoleic acid supplementation [66] were both found to alter expression of DNMT1, as well as MeCP2, a methyl binding protein that binds methylated DNA and recruits additional transcriptional modifiers. DNA demethylation can also alter gene expression, whether by direct or indirect mechanisms such as changes in base excision repair or decreasing DNMT levels. One example is that folate depletion during pregnancy can increase base excision repair (BER) in offspring, but during weaning, BER falls and methylation changes in the DNA occur. Such changes can also increase oxidative stress and predispose the child to neurological disorders later in life [67].

Chronic substance abuse can lead to malnutrition and micronutrient deficiency [68]. In a mouse model, ingestion of ethanol results in hippocampal DNA methylation alterations during development [69]. In humans, there is evidence that folate depletion during pregnancy can also be a result of small doses of methanol found in alcohol [70]. Transcriptional and epigenetic changes as a result of these micronutrient deficiencies from substance abuse are another way through which methylation and demethylation may cause psychopathology during development.

In humans, there is strong evidence from analyses of both the Dutch Hunger Winter and the Chinese famine (1959–1961) showing a correlation between pre-natal famine exposure and schizophrenia [71,72,73]. Subsequently, investigations of epigenetic differences have been initialed. Retrospective studies investigating the effect of the Dutch Hunger Winter (1944–1945) have found that exposure to famine during pregnancy is linked to hypo- and hyper- methylation in certain regions of DNA, when compared to same sex, unexposed siblings [74,75]. Another study by Lumey *et al.* [76] found that there was no significant correlation between pre-natal famine exposure and global DNA methylation in the sample of adults conceived during the Dutch Hunger Winter, however differential methylation at specific loci was demonstrated [77].

Furthermore, epigenetic regulation can be a result of histone modifications as well as DNA methylation [78]. Histone methylations are a mechanism for chromatin remodeling and certain histone modifications, such as acetylation or ubiquitination, can also silence or activate certain genes or allow for DNA methylation and demethylation [79].

#### 4.1.2. Influence of Epigenetic Changes on Brain Dysfunction and Psychopathology

As described above, a number of studies have made the connection between nutrition deficiency and epigenetic changes apparent. Animal studies provide further evidence that malnutrition during early life (gestation and lactation) can alter DNA methylation within the brain. Mouse models of protein restriction during early life have shown both global [65] and promoter-specific [80,81] decreases in DNA methylation. Beyond protein, iron deficiency in early life, a well characterized risk factor for impaired cognitive development [82], has also been shown to reduce DNA methylation in the brain [83].

Similarly, there is strong evidence supporting the connection between specific epigenetic changes in neurons and resulting gene expression changes. For example, a cell culture study by Chen *et al.* [84] found that methylation of the promoter region of *reelin*, a protein involved in neuronal development and synaptogenesis, was correlated with reduction of its expression in the prefrontal cortex, a region of the brain which is tied to impulse control, cognitive behaviors, and personality expression. Differential nutrient availability during the prenatal and neonatal periods has also been known to lead to long-lasting changes in neuron development. For example, in a cell culture study by Niculescu *et al.* [85], when pregnant rodents were fed a choline deficient diet, the *CDKN3* gene promoter was hypomethylated in the fetal brain, resulting in an over-expression of the gene, leading to decreased neuroblastoma cell proliferation. Additionally, early life protein restriction was found to drive significant transcriptional changes in the prefrontal cortex, as well impaired performance in an attentional task [86]. Interestingly, performance deficits were found to be correlated with increased DNMT expression. However, while it may seem intuitive to conclude that the reduced availability of methylation precursors leads to lower levels of DNA methylation, there is evidence that the relationship is not that simple. In the Pogribny *et al.* [59] study mentioned above, neurons from rats fed a folate-deficient diet were found to have global as well as gene-specific DNA hypermethylation. Further studies are needed to clearly delineate the cellular mechanisms that connect dietary methyl-deficiency to differential DNA methylation.

Specific epigenetic changes in brain cells have also been correlated with psychopathologies such as depression, addiction, and schizophrenia [78]. Yet there is a lack of studies connecting a specific nutrition deficiency to a particular epigenetic change, while also connecting that to a specific psychopathology. However, there are studies that have connected a specific toxin exposure to a particular epigenetic change and a resulting psychopathology. For example, mice exposed perinatally to methylmercury were found to have a number of epigenetic alterations, including DNA hypermethylation, in the *BDNF* promoter region in hippocampal cells, resulting in suppression of BDNF gene expression in those cells, which was then found to induce depression-like behavior in mice [87].

### 4.2. Altered Brain Structure and Function as a Mediator

In the proposed framework, altered brain structure and function acts as a mediator through which nutrition deficiency can cause behavior problems and psychopathology through three main routes: impaired brain development, signaling molecule imbalance, and increased neurotoxicity of heavy metals. This mediation can either be precipitated directly (*i.e.*, nutrition deficiency directly causes brain dysfunction) or indirectly through epigenetic changes (*i.e.*, nutrition deficiency causes epigenetic changes, which then cause brain dysfunction), as was addressed in the above section.

#### 4.2.1. Impaired Brain Development

In maintaining normal structure and function of the central nervous system, both protein and micronutrients are known to play essential roles [14,88]. Animal studies in the past have found evidence that nutrition deficiency during early life reduces the growth of the brain and permanently decreases brain size and cellular content [89].

For instance, dietary protein has been shown to be instrumental in early body and brain development. Gressens [90] found that rats that were introduced to dietary protein restriction during pregnancy produced offspring who were significantly smaller in body size and in brain cortical areas compared to controls. More recently, Lucassen *et al.* [91] have found that nutritional stress during gestation or lactation alters hippocampal structure and cognition.

Iron and zinc have also been shown to be critical to early brain development, as they are essential for the synthesis and maintenance of myelin content in the central and peripheral nervous systems. Myelination of a neuron’s axon vastly increases the speed and coordination of electrical impulse transmission down the axon. Consequently, deficiency in iron and zinc can lead to alterations in brain growth, development, and function. Other studies on rats have indicated that supplementation of both zinc and iron help accelerate recovery of hippocampal function following periods of iron deficiency [92]. In a study with Bangaladeshi infants, dietary supplementation with zinc and iron was shown to promote motor development and exploratory behavior [93].

Biochemical evidence has shown that docosahexaenoic acid (DHA), an omega-3 fatty acid, is the richest fatty acid in the brain. DHA is the critical building block for gray matter and plays a key role in the biochemical functions of the brain. DHA therefore plays an essential role in the development of the fetal brain, particularly during the first few months of pre-natal life when there is rapid growth [94]. Brain dysfunctions caused by omega-3 nutrition deficiency has also been implicated in specific psychopathologies, such as the pathophysiology of aggressive disorders in humans [15]. There is evidence that choline supplementation in the prenatal diet can potentially program, through epigenetic mechanisms, expression of growth factors and hippocampal cell proliferation [95], while DHA can have similar effects on and alterations in neurite growth [96].

Because of the intimate connection between these various nutrient deficiencies and brain development and maintenance, it follows that nutrition deficiency may directly cause brain deficits by reducing brain cell growth and development, which then in turn predispose violent and criminal behavior [7]. The brain deficits caused by nutrition deficiency can also manifest in more subtle ways, such as impairments in cognitive functioning, which are also closely correlated with behavioral problems [91,97]. Low intelligence (IQ) has been found to be a mediating factor in the relationship between nutrition deficiency and increased externalizing behavior throughout childhood and adolescence [6,8].

#### 4.2.2. Signaling Molecule Imbalance

Micronutrients play an important role in influencing neurotransmission because the function of the brain is fundamentally related to its metabolism of nutrients [98]. Neurotransmitter metabolism, in turn, involves a chain of biochemical processes, which rely on vitamins and minerals that function as co-enzymes in every step of neurotransmission including neurotransmitter production, release, inhibition, transmission, and receptor formation.

The neurotransmitter impairments implicated in impulsive and aggressive behavior most regularly involved serotonin and dopamine. Tryptophan, the essential amino acid precursor to serotonin, has been linked directly to brain levels of serotonin [12]. A functional Magnetic Resonance Imaging (MRI) study by Rubia *et al.* [16] in humans reveals that tryptophan depletion produced by a change in diet reduces right inferior prefrontal activation during a response inhibition task, a task which required subjects to inhibit an inappropriate response to a stimulus. These results suggest that a disruption of tryptophan levels in adults may precipitate acute psychopathology, as reduced prefrontal activation has been linked to antisocial behavior [99]. Other studies have also shown that prenatal deficiency of omega-3 fatty acid in rats results in decreased density of synaptic vesicles at the terminal ends of neurons [100] and can negatively impact serotonin transmission [101]. Further, prenatal protein deficiency has been repeatedly shown to adversely affect development of the dopamine system [80,102,103,104], as well as dopamine-related behaviors, such as a decrease in social behavior, increased anxiety, and increased locomotor activity [65,80,105]. Both animal and human studies have repeatedly linked aggression to lower brain levels of serotonin [29,106,107].

Similarly, the bioavailability of iron in the brain has been shown to affect neurotransmitter production and function in the dopamine-opiate systems of the brain. Animal studies have shown that iron deficiency may alter behavior by reducing dopamine transmission [108]. Zimmer *et al.* [109] found that rats deficient in omega-3 fatty acids exhibited altered dopamine neurotransmission. There is also evidence that zinc is a key co-factor for building up neurotransmitter and fatty acids and is indirectly involved in the metabolism of dopamine and fatty acids, which consequently affects behavior [110].

Animal studies have indicated that protein deficiency during pregnancy can induce a significant decrease in the activity of brain monoamine oxidase (MAO) compared to controls. Another animal study found that there is a relationship between aggressiveness and low MAO-A activity through the elevation of brain levels of serotonin, norepinephrine, and dopamine [111].

In humans, low MAO-B activity has been reported to be linked to aggression, impulsiveness, and sensation-seeking behavior in psychiatric evaluations of adult males [112,113]. Caspi *et al.* [114] further confirmed that maltreated children with a genotype conferring high levels of MAO-A expression were less likely to develop antisocial problems.

#### 4.2.3. Increasing Neurotoxicity

A growing area of study shows that there are genetic factors that affect how environmental toxicants influence health outcomes. One example of a gene linked with lead poisoning and epigenetic regulation is the *ALAD* gene [115]. Methylation of the *ALAD* gene promoter has been found to play an important role in increasing or decreasing risk for lead poisoning, which has been well-recognized as a neurotoxicant. Increased *ALAD* gene methylation was found to decrease gene transcription, and made individuals more susceptible to lead toxicity [116]. Furthermore, a study of Bangladeshi children found that there were different *ALAD* polymorphisms that had varying effects on how blood lead levels impact an individual’s health [117]. The interaction of environmental toxicants and genetics can determine behavioral and psychological health outcomes and the severity of such health effects.

In recent years, there has been increased attention on the role of metal toxicity in brain development and behavior. Research has found that prenatal lead exposure is related to reduced total brain volume [118] and postnatal lead exposure can potentially have deleterious effects on neural progenitor cell proliferation and therein negatively affect the structure and function of the hippocampus [119]. Animal studies on rats have also found that microinjection of manganese chloride can cause neurodegenerative processes that can further alter the animals’ emotional behavior [120]. Excessive copper in the neonatal brain is also associated with abnormal development of the hippocampus, the portion of the brain which is critical in learning and which has been shown to function abnormally in murderers [50,121].

However, studies have shown that the individual effects of some neurotoxins do not directly cause behavior problems. Rather, the effects are magnified only when coupled with nutritional deficits such as protein or calcium deficiency. Masters *et al.* [50], have reported that animals on a diet high in manganese do not exhibit high levels of blood manganese when the diet also contained normal levels of calcium. Lead’s ability to substitute for calcium and perhaps zinc is believed to be a factor common to many of its toxic actions. We recently found that regular breakfast consumption reduces blood lead levels in children, which provides initial evidence of some protective effect of nutrition in lead-exposed children [6].

In recent years, the association between lead exposure and aggression has been receiving increased attention, with evidence accumulating from experimental research in animals [122], epidemiological studies in community children [52,123], as well as in juveniles delinquents [124] and criminal offenders [125]. Even at low mean blood lead levels of 6.4 µg/dL, lead exposure is still associated with internalizing and externalizing behavior [52].

Although the mechanisms by which neurotoxins induce aggressive behavior are not yet fully understood, research has revealed that neurotoxins are involved in neurotransmission processes. Murphy *et al.* [126] found that levels of dopamine, norepinephrine, and serotonin were lowered in manganese intoxicated animals. Furthermore, rats exposed to high lead levels exhibited inhibition of the NMDA receptor, which plays a critical role in learning and conditioning [127]. As discussed earlier in Figure 1, epigenetic changes can affect the expression of certain genes that code for proteins affecting neurotransmitters. Collectively, it is possible that interaction among epigenetics, nutrition, and neurotoxicants, which further affects brain function can impact psychopathologies.

## 5. Conclusions

The proposed framework illustrates an interactive mechanism by which diet and nutrition can lead to behavioral outcomes such as aggression, delinquency, hyperactivity, and anti-social behavior. While it has been previously proposed that brain dysfunction plays an integral role in mediating nutrition deficiency and psychopathology [11], the role of the epigenome as another link between nutrition and behavior has received attention only very recently. While historically, genomic risk factors for violence and aggression have been viewed as somewhat elusive to intervention, the inclusion of epigenetic mechanisms provides a pathway by which the biological and potentially hereditary risks for psychopathology can mitigated e.g., via proper nutrition. As Szyf [128] points out, unlike genetic mechanisms, epigenetic mechanisms are dynamic and therefore potentially reversible by interventions.

There is ample evidence in the literature that epigenetic alterations affect brain development and neurological function, both directly by influencing the anatomical structure of the brain and indirectly by altering the chemical environment and endocrine balance of the central nervous system [84,109,129,130]. The epigenome is, therefore, an important area of future studies of prevention of psychopathology because it is in constant and dynamic equilibrium with its environment and therefore suitable for diet and nutrition to act upon. Nutritional excess and obesity as well as nutrition deficiency can affect the epigenome in ways that we have not explored in this review paper. This could also be a cause of psychopathology based on the equilibrium between environment and genes [131].

There are a number of studies that explore the biological and psychosocial risk factors for adverse behavioral outcomes [6,132,133] with emphasis on the early risk factors (e.g., during the pre- and peri-natal periods and early childhood). However, to fully flesh out the relationship between nutrition and psychopathology, there is a need for more studies focused on linking together nutrition deficiency, epigenetic changes, and the resulting brain dysfunction. The inclusion of epigenetic mechanisms can potentially expand the application of the framework further into later stages in life because nutrition and diet can impact brain function across a lifespan via epigenetics. A better understanding of the mechanisms underlying the complex interactions between nutrition deficiency, brain dysfunction, epigenetics, and adverse behavioral outcomes can potentially help the development of effective primary prevention and intervention programs and mitigate the nutritional risk factors of psychopathology. Furthermore as Hubbs-Tait *et al.* [47] points out, behavior has various, complex influences, particularly with regard to children’s development. Nutrition, social environment, and neurotoxicants can all contribute to behavior, and the nuances in behavioral development need to continually be investigated.
